# Supplementary material for: Hot spot prediction in protein-protein interactions by an ensemble system
Source: BMC Syst Biol. 2018 Dec 31;12(Suppl 9):132. doi: 10.1186/s12918-018-0665-8 (PMC6311905; doi:10.1186/s12918-018-0665-8)
Supplement: Supplementary file 2 — All relevant MATLAB code. A simple Matlab implement of our predictor. (ZIP 19,742 kb) [file 12918_2018_665_MOESM2_ESM.zip › Additional file 2/Read ASA/ASA website/ASEdb/1GC1C.htm]

 NetSurfP - Protein Surface Accessibility and Secondary Structure Predictions

  

|  |  |  |
| --- | --- | --- |
|  |  | NetSurfP - Protein Surface Accessibility and Secondary Structure Predictions  Technical University of Denmark |

  


---

```
# For publication of results, please cite:
# A generic method for assignment of reliability scores applied to solvent accessibility predictions.
# Bent Petersen, Thomas Nordahl Petersen, Pernille Andersen, Morten Nielsen and Claus Lundegaard
# BMC Structural Biology 2009, 9:51 doi:10.1186/1472-6807-9-51
#
# Column 1: Class assignment - B for buried or E for Exposed - Threshold: 25% exposure, but not based on RSA
# Column 2: Amino acid
# Column 3: Sequence name
# Column 4: Amino acid number
# Column 5: Relative Surface Accessibility - RSA
# Column 6: Absolute Surface Accessibility
# Column 7: Z-fit score for RSA prediction
# Column 8: Probability for Alpha-Helix
# Column 9: Probability for Beta-strand
# Column 10: Probability for Coil
E K  1GC1C                  1    0.747 153.596   1.313   0.003   0.003   0.994
E K  1GC1C                  2    0.616 126.711   0.768   0.004   0.514   0.481
E V  1GC1C                  3    0.424  65.200   1.300   0.001   0.900   0.099
B V  1GC1C                  4    0.099  15.216   0.595   0.001   0.959   0.040
E L  1GC1C                  5    0.380  69.560   1.598   0.001   0.959   0.040
B G  1GC1C                  6    0.068   5.375  -0.089   0.001   0.959   0.040
E K  1GC1C                  7    0.462  95.013   1.744   0.001   0.959   0.040
E K  1GC1C                  8    0.461  94.848   1.060   0.004   0.514   0.481
E G  1GC1C                  9    0.642  50.510  -0.574   0.005   0.045   0.951
E D  1GC1C                 10    0.566  81.503   1.178   0.004   0.085   0.910
E T  1GC1C                 11    0.529  73.386   1.545   0.003   0.718   0.279
B V  1GC1C                 12    0.047   7.178   0.408   0.001   0.959   0.040
E E  1GC1C                 13    0.362  63.241   1.900   0.001   0.959   0.040
B L  1GC1C                 14    0.046   8.423   0.890   0.001   0.959   0.040
E T  1GC1C                 15    0.366  50.778   1.628   0.001   0.959   0.040
B C  1GC1C                 16    0.025   3.538   0.525   0.001   0.900   0.099
E T  1GC1C                 17    0.457  63.400   1.399   0.001   0.959   0.040
B A  1GC1C                 18    0.072   7.978  -2.017   0.001   0.900   0.099
E S  1GC1C                 19    0.584  68.445   0.144   0.004   0.420   0.576
E Q  1GC1C                 20    0.734 131.128  -1.671   0.005   0.045   0.951
E K  1GC1C                 21    0.493 101.410  -0.624   0.005   0.015   0.979
E K  1GC1C                 22    0.573 117.907  -1.074   0.005   0.045   0.951
E S  1GC1C                 23    0.591  69.300   0.500   0.004   0.085   0.910
B I  1GC1C                 24    0.121  22.404   0.390   0.005   0.336   0.660
E Q  1GC1C                 25    0.397  70.958   1.441   0.002   0.816   0.182
B F  1GC1C                 26    0.040   8.048   0.296   0.001   0.959   0.040
E H  1GC1C                 27    0.270  49.040   1.344   0.001   0.959   0.040
B W  1GC1C                 28    0.039   9.283  -0.142   0.001   0.959   0.040
E K  1GC1C                 29    0.326  67.099   1.003   0.001   0.959   0.040
E N  1GC1C                 30    0.234  34.199   0.740   0.003   0.718   0.279
E S  1GC1C                 31    0.639  74.914  -0.671   0.005   0.045   0.951
E N  1GC1C                 32    0.743 108.746  -1.552   0.005   0.015   0.979
E Q  1GC1C                 33    0.588 105.017  -0.751   0.004   0.420   0.576
E I  1GC1C                 34    0.495  91.575  -0.376   0.003   0.718   0.279
E K  1GC1C                 35    0.319  65.598   0.326   0.021   0.756   0.223
E I  1GC1C                 36    0.268  49.561  -0.351   0.021   0.756   0.223
B L  1GC1C                 37    0.269  49.272  -0.850   0.022   0.552   0.426
E G  1GC1C                 38    0.470  36.973  -1.407   0.020   0.205   0.775
E N  1GC1C                 39    0.428  62.703  -1.809   0.019   0.141   0.840
E Q  1GC1C                 40    0.555  99.177  -1.438   0.020   0.205   0.775
E G  1GC1C                 41    0.484  38.051  -1.611   0.021   0.279   0.699
E S  1GC1C                 42    0.465  54.533  -0.539   0.023   0.655   0.322
B F  1GC1C                 43    0.256  51.379  -0.931   0.021   0.756   0.223
E L  1GC1C                 44    0.293  53.703  -0.256   0.018   0.846   0.136
E T  1GC1C                 45    0.360  49.960  -1.124   0.021   0.756   0.223
E K  1GC1C                 46    0.509 104.681  -0.849   0.021   0.451   0.528
E G  1GC1C                 47    0.508  39.995  -1.650   0.021   0.279   0.699
E P  1GC1C                 48    0.550  78.102  -1.145   0.020   0.205   0.775
E S  1GC1C                 49    0.485  56.819  -1.346   0.021   0.279   0.699
E K  1GC1C                 50    0.564 116.118  -0.631   0.021   0.279   0.699
E L  1GC1C                 51    0.338  61.943  -1.026   0.066   0.296   0.638
E N  1GC1C                 52    0.520  76.201  -1.245   0.021   0.279   0.699
E D  1GC1C                 53    0.421  60.637  -1.701   0.021   0.279   0.699
E R  1GC1C                 54    0.336  76.967  -1.015   0.021   0.451   0.528
B A  1GC1C                 55    0.234  25.809  -1.488   0.022   0.552   0.426
E D  1GC1C                 56    0.460  66.315  -1.014   0.022   0.552   0.426
B S  1GC1C                 57    0.272  31.843  -1.438   0.022   0.552   0.426
E R  1GC1C                 58    0.383  87.707  -0.915   0.022   0.552   0.426
E R  1GC1C                 59    0.493 112.989  -0.977   0.021   0.451   0.528
E S  1GC1C                 60    0.528  61.940  -0.929   0.021   0.451   0.528
E L  1GC1C                 61    0.406  74.320  -1.120   0.022   0.359   0.619
E W  1GC1C                 62    0.369  88.624  -1.203   0.022   0.359   0.619
E D  1GC1C                 63    0.590  84.990  -1.489   0.022   0.359   0.619
E Q  1GC1C                 64    0.494  88.228  -1.554   0.004   0.138   0.858
E G  1GC1C                 65    0.517  40.719  -1.671   0.004   0.138   0.858
E N  1GC1C                 66    0.532  77.885  -0.657   0.005   0.262   0.733
B F  1GC1C                 67    0.100  20.150  -0.066   0.004   0.616   0.381
B P  1GC1C                 68    0.220  31.190   0.582   0.001   0.900   0.099
B L  1GC1C                 69    0.020   3.680   0.962   0.001   0.959   0.040
E I  1GC1C                 70    0.256  47.397   1.568   0.001   0.959   0.040
B I  1GC1C                 71    0.027   4.940   0.412   0.001   0.900   0.099
E K  1GC1C                 72    0.448  92.112   1.590   0.002   0.816   0.182
E N  1GC1C                 73    0.528  77.270   0.396   0.004   0.616   0.381
B L  1GC1C                 74    0.137  25.066   0.526   0.005   0.336   0.660
E K  1GC1C                 75    0.492 101.266   1.069   0.005   0.336   0.660
E I  1GC1C                 76    0.557 103.082   0.145   0.005   0.336   0.660
E E  1GC1C                 77    0.647 113.118   0.220   0.004   0.138   0.858
B D  1GC1C                 78    0.255  36.760  -0.855   0.004   0.085   0.910
E S  1GC1C                 79    0.566  66.394  -0.278   0.004   0.138   0.858
B D  1GC1C                 80    0.244  35.088  -1.187   0.004   0.616   0.381
E T  1GC1C                 81    0.426  59.156   1.958   0.001   0.900   0.099
B Y  1GC1C                 82    0.059  12.501   0.680   0.001   0.959   0.040
E I  1GC1C                 83    0.281  51.929   2.123   0.001   0.959   0.040
B C  1GC1C                 84    0.018   2.457   1.263   0.001   0.959   0.040
E E  1GC1C                 85    0.300  52.323   1.927   0.001   0.959   0.040
B V  1GC1C                 86    0.027   4.165   0.095   0.001   0.959   0.040
E E  1GC1C                 87    0.485  84.747   0.759   0.002   0.816   0.182
E D  1GC1C                 88    0.395  56.963  -0.276   0.004   0.420   0.576
E Q  1GC1C                 89    0.615 109.893   0.017   0.004   0.197   0.799
E K  1GC1C                 90    0.650 133.787   0.145   0.005   0.262   0.733
E E  1GC1C                 91    0.471  82.266  -0.438   0.003   0.718   0.279
E E  1GC1C                 92    0.457  79.855   0.750   0.001   0.900   0.099
B V  1GC1C                 93    0.133  20.442  -0.453   0.001   0.900   0.099
E Q  1GC1C                 94    0.350  62.492   0.629   0.001   0.900   0.099
B L  1GC1C                 95    0.104  19.097  -0.186   0.001   0.900   0.099
B L  1GC1C                 96    0.238  43.651   0.753   0.001   0.959   0.040
B V  1GC1C                 97    0.192  29.572  -0.238   0.001   0.900   0.099
B F  1GC1C                 98    0.201  40.260   0.371   0.001   0.959   0.040
E G  1GC1C                 99    0.521  40.979   0.353   0.001   0.900   0.099
B L  1GC1C                100    0.093  17.065  -0.157   0.002   0.816   0.182
E T  1GC1C                101    0.566  78.504   0.150   0.004   0.616   0.381
E A  1GC1C                102    0.271  29.908   0.038   0.005   0.262   0.733
E N  1GC1C                103    0.455  66.685  -0.285   0.005   0.045   0.951
E S  1GC1C                104    0.537  62.925  -1.279   0.005   0.015   0.979
E D  1GC1C                105    0.689  99.256  -0.174   0.004   0.085   0.910
B T  1GC1C                106    0.227  31.429   0.667   0.004   0.514   0.481
E H  1GC1C                107    0.522  95.043   0.848   0.002   0.816   0.182
B L  1GC1C                108    0.114  20.855   0.594   0.001   0.900   0.099
E L  1GC1C                109    0.454  83.091   1.234   0.003   0.718   0.279
E Q  1GC1C                110    0.520  92.908   0.578   0.005   0.262   0.733
E G  1GC1C                111    0.634  49.880  -0.865   0.005   0.015   0.979
E Q  1GC1C                112    0.451  80.477   0.857   0.005   0.045   0.951
E S  1GC1C                113    0.518  60.721   1.156   0.004   0.514   0.481
B L  1GC1C                114    0.056  10.180   0.665   0.001   0.900   0.099
E T  1GC1C                115    0.398  55.258   1.974   0.001   0.959   0.040
B L  1GC1C                116    0.052   9.430   0.965   0.001   0.959   0.040
E T  1GC1C                117    0.355  49.197   1.820   0.001   0.959   0.040
B L  1GC1C                118    0.022   4.083   0.824   0.001   0.959   0.040
E E  1GC1C                119    0.483  84.310   1.446   0.001   0.959   0.040
B S  1GC1C                120    0.074   8.685  -1.363   0.001   0.900   0.099
E P  1GC1C                121    0.594  84.232   0.460   0.004   0.616   0.381
E P  1GC1C                122    0.521  73.902  -1.845   0.004   0.138   0.858
E G  1GC1C                123    0.567  44.591  -0.348   0.005   0.045   0.951
B S  1GC1C                124    0.224  26.300  -0.624   0.005   0.045   0.951
E S  1GC1C                125    0.548  64.249   0.892   0.004   0.138   0.858
B P  1GC1C                126    0.099  13.991  -0.232   0.004   0.514   0.481
E S  1GC1C                127    0.418  48.943   1.102   0.001   0.900   0.099
B V  1GC1C                128    0.043   6.563   0.048   0.001   0.959   0.040
E Q  1GC1C                129    0.314  56.080   1.518   0.001   0.959   0.040
B C  1GC1C                130    0.037   5.125  -0.176   0.011   0.918   0.071
E R  1GC1C                131    0.329  75.295   1.008   0.001   0.900   0.099
E S  1GC1C                132    0.224  26.288   0.835   0.004   0.616   0.381
E P  1GC1C                133    0.667  94.605  -0.078   0.005   0.015   0.979
E R  1GC1C                134    0.764 174.887  -1.360   0.003   0.003   0.994
E G  1GC1C                135    0.620  48.778  -0.572   0.004   0.420   0.576
E K  1GC1C                136    0.605 124.469   0.430   0.003   0.718   0.279
E N  1GC1C                137    0.350  51.284   0.130   0.021   0.756   0.223
E I  1GC1C                138    0.408  75.480   0.068   0.004   0.616   0.381
E Q  1GC1C                139    0.546  97.587  -0.968   0.005   0.336   0.660
E G  1GC1C                140    0.784  61.693  -0.867   0.004   0.085   0.910
E G  1GC1C                141    0.711  55.971  -0.732   0.005   0.045   0.951
E K  1GC1C                142    0.397  81.642   0.118   0.005   0.262   0.733
E T  1GC1C                143    0.399  55.314   1.228   0.002   0.816   0.182
B L  1GC1C                144    0.037   6.701  -0.164   0.001   0.959   0.040
E S  1GC1C                145    0.336  39.379   1.896   0.001   0.959   0.040
B V  1GC1C                146    0.050   7.624   0.234   0.001   0.900   0.099
E S  1GC1C                147    0.447  52.424   1.531   0.002   0.816   0.182
E Q  1GC1C                148    0.566 101.123   1.044   0.004   0.616   0.381
B L  1GC1C                149    0.088  16.076   0.269   0.004   0.420   0.576
E E  1GC1C                150    0.462  80.746   1.060   0.004   0.514   0.481
E L  1GC1C                151    0.514  94.150   0.315   0.004   0.420   0.576
E Q  1GC1C                152    0.606 108.160  -0.445   0.004   0.197   0.799
B D  1GC1C                153    0.276  39.728  -0.498   0.004   0.085   0.910
E S  1GC1C                154    0.485  56.854  -0.573   0.004   0.138   0.858
B G  1GC1C                155    0.152  11.939  -1.557   0.002   0.816   0.182
E T  1GC1C                156    0.411  56.964   1.636   0.001   0.900   0.099
B W  1GC1C                157    0.064  15.344   0.676   0.001   0.959   0.040
E T  1GC1C                158    0.276  38.253   1.608   0.001   0.959   0.040
B C  1GC1C                159    0.018   2.499   1.226   0.001   0.959   0.040
E T  1GC1C                160    0.259  35.868   1.962   0.001   0.959   0.040
B V  1GC1C                161    0.040   6.209  -0.364   0.001   0.959   0.040
E L  1GC1C                162    0.396  72.544   0.408   0.001   0.959   0.040
B Q  1GC1C                163    0.142  25.433   0.034   0.004   0.514   0.481
E N  1GC1C                164    0.729 106.682   0.228   0.005   0.045   0.951
E Q  1GC1C                165    0.521  93.015  -0.693   0.004   0.085   0.910
E K  1GC1C                166    0.538 110.749  -0.630   0.005   0.336   0.660
E K  1GC1C                167    0.498 102.397  -0.054   0.002   0.816   0.182
E V  1GC1C                168    0.305  46.894   0.110   0.001   0.900   0.099
E E  1GC1C                169    0.484  84.572   0.323   0.001   0.959   0.040
B F  1GC1C                170    0.194  38.856   0.785   0.001   0.959   0.040
E K  1GC1C                171    0.413  84.954   0.774   0.001   0.959   0.040
B I  1GC1C                172    0.048   8.843   0.423   0.001   0.959   0.040
E D  1GC1C                173    0.345  49.758   1.708   0.001   0.959   0.040
B I  1GC1C                174    0.026   4.791   0.767   0.001   0.959   0.040
E V  1GC1C                175    0.255  39.240   1.856   0.001   0.959   0.040
B V  1GC1C                176    0.045   6.855   0.088   0.001   0.959   0.040
E L  1GC1C                177    0.296  54.124   0.751   0.018   0.846   0.136
E A  1GC1C                178    0.346  38.173  -0.570   0.023   0.655   0.322
E F  1GC1C                179    0.350  70.185   0.093   0.004   0.197   0.799
E Q  1GC1C                180    0.409  72.976  -0.172   0.005   0.045   0.951
E K  1GC1C                181    0.702 144.360   0.097   0.005   0.015   0.979
E A  1GC1C                182    0.270  29.721  -0.985   0.005   0.015   0.979
E S  1GC1C                183    0.534  62.561  -1.385   0.005   0.015   0.979
E N  1GC1C                184    0.677  99.084  -1.849   0.018   0.047   0.935
E T  1GC1C                185    0.705  97.811  -1.670   0.003   0.003   0.994

Explain the output.  Go back.
```

---
